# *N-tert*-Butoxycarbonylation of Structurally Diverse Amines and Sulfamides under Water-Mediated Catalyst-Free Conditions

**DOI:** 10.5402/2012/404235

**Published:** 2012-05-09

**Authors:** Zinelaabine Cheraiet, Souad Ouarna, Sihem Hessainia, Malika Berredjem, Nour-Eddine Aouf

**Affiliations:** LCOA, Bioorganic Chemistry Group, Chemistry Department, Sciences Faculty, Badji Mokhtar-Annaba University, P.O. Box. 12, 23000 Annaba, Algeria

## Abstract

A simple, efficient, and eco-friendly protocol for the *N*-Boc protection of the amine moiety in a variety of compounds with di-*tert*-butyl dicarbonate under water-acetone catalyst-free conditions is described. The corresponding monocarbamate is obtained in excellent yields on short reaction times. No competitive side reactions such as isocyanate urea and O-Boc were observed. This method represents a reasonable alternative to the previous reported protection procedures.

## 1. Introduction

The protection of a functional group can be essential in the chemistry of poly functionalised molecules, when a reaction has to be carried out in a part of the compounds without the rest perturbing of the molecule. The development of simple and eco-friendly methods for the protection and deprotection of functional group continues to be a significant tool in synthetic chemistry of polyfunctional molecules [1, 2]. 

Nitrogen protection continues to attach a great deal of attention in a wide range of chemical fields, such as peptides, nucleosides, heterocyclic compounds, and other natural products. The protection of amines with *tert*-butyloxycarbonyl (Boc) group is a widely used reaction in organic synthesis because of its inertness toward catalytic hydrogenolysis and resistance toward hydrolysis under most basic conditions and nucleophilic reagents [3]. *N*-Boc deprotection is generally achieved under mild acidic conditions such as trifluroacetic acid (TFA), aqueous phosphoric acid in THF [4], or Lewis acid [5]. The deprotection can be carried out with montmorillonite K.10 clay [6], silica gel at low pressure [7], and by thermolytic cleavage although at high temperature [8, 9].

The *tert*-butyloxycarbonyl (Boc) is easily introduced using commercially available di-*tert-*butyldicarbonate (*tert*-BuOCO)_2_O under standard basic conditions. Various reagents and methods have been developed in the last years for the *N*-*tert*-butyloxycarbonylation of amines. Most are carried out in the presence of an organic or inorganic base. Amines are converted to *N-tert-*Boc derivatives by reaction with di-*tert-*butyldicarbonate (Boc)_2_O in the presence of: 4-(dimethylamino)-1-*tert*-butylcarbonylpyridinium DMAP [10], 4-(dimethylamino)-1-*tert*-butylcarbonyl pyridinium chloride [11] or tetrafluoroborate in aq NaOH [12], *tert*-butyl-2-pyridyl carbonate in the presence of K_2_CO_3_ in H_2_O-DMF [13] or *tert*-butyl 1-chloroethyl carbonate in presence of K_2_CO_3_ in H_2_O-THF [14], 2-*tert*-butyloxycarbonyloxyimino-2-phenylacetonitrile in the presence of Et_3_N in H_2_O-dioxane [15]. However, these protocols have various drawbacks as long times, preparation of *tert*-butoxycarbonylation reagents, and requirement of auxiliary substances.

The base-catalyzed reactions are often associated with the formation of isocyanate [16], urea [10], and *N*, *N*-di-Boc derivatives [17]. Moreover, the high toxicity of DMAP and reagents derived from it limits their use [18]. The protection can also be affected with mild acidic conditions. There are examples of other modified methods for *ter*-butoxycarbonylation of amines with H_3_PW_12_O_40_ [19], Amberlyst 15 [20], Guanidine hydrochloride [21], Zn (ClO_4_)·6H_2_O [22], ZrCl_4 _[23], LiClO_4_ [24], Cu (BF_4_)_2_ [25], sulfonic acid functionalized silica [26], and HClO_4_-SiO_2_ [27]. 

More recently, Akbari et al. reported an efficient protocol for the *N*-protection of various structurally amines using protic 1, 2, 3, 3-*tetra*-methyguanidinium acetate as recyclable catalyst under solvent free condition at room temperature [28]. Many of these methods suffer from disadvantages such high acidity, expensive reagents, and using more excess. Excessive amounts of catalysts, high temperature and slow rate reaction. Chankeshwara and Chakraborti [29] reported the catalyst-free chemoselective *N*-*tert*-butyloxycarbonylation of amines in water. This method is not reproducible because the limited solubility of (Boc)_2_O in water under ambient conditions.

In recent years, much attention has been focused on searching greener or environmentally friendly chemical process. Water is the main solvent for life processes, and there is growing interest in using it as green solvent for organic transformations [30, 31]. However, reports about using water as a catalyst to promote organic reactions are very limited. Compared to conventional solvents water is preferred for organic reaction because it displays unparalleled and unique properties. Moreover, it is cheap, nontoxic, nonexplosive, and environmentally acceptable [32, 33]. Thus, the use of water instead of organic solvents has gained much importance in the development of sustainable protection in generally chemistry.

In this paper, we report efficient and eco-friendly protocol for chemoselective *N*-*tert*-butyloxycarbonylation of various structurally amines in water-related system under ambient conditions in the absence of any acid/base-catalyst.

## 2. Results and Discussion

**Scheme 1:**
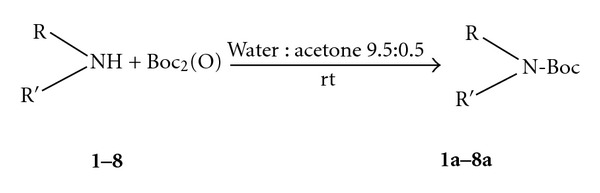


In our quest of a “greener” approach toward *N*-Boc protection, we have carried out a series of experiments using commercially available di-*tert*-butyldicarbonate (*tert*-BuOCO)_2_O and various structurally amines and water as solvent (Scheme 1). The *N*-*tert*-butyloxycarbonylation of various amines (Table 1) was carried out in distilled water with a minimum of aceton at room temperature and atmospheric pressure in the absence of any catalyst (Scheme 1).

The reactions were completed after 8–12 min, affording Boc protected amines in good and excellent yields (Table 1) and short time. In each case, only the mono *N*-Boc protected product was found. No isocyanate or urea formation was detected (by NMR of crude products). 

The critical amount of water required was found to be 1 mL/mmol of amine and the minimum of the aceton for the solubility of (Boc)_2_O. The products were isolated by filtration (for solid products) or extraction with CH_2_Cl_2_ (for liquid products).

The chemoselectivity was further demonstrated in the case of p-aminophenol (entry **5**) that did not form oxazolidinone.

To explore the scope and limitations of this reaction and view of the importance of peptide synthesis, we investigated the Boc-protection of various aminoesters derivatives of (Leu, Ala, Val, Leu, and Phe) (Table 2, entries **9**–**13**).

**Scheme 2:**
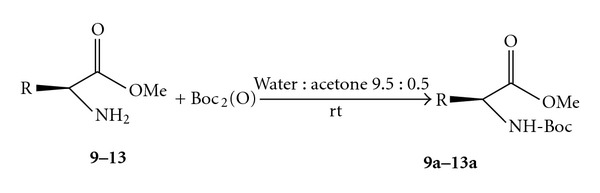


All *N*-Boc-protected aminoesters were prepared from the corresponding starting from aminoacids after esterification and protection by reacting with (Boc)_2_O in water at room temperature (Scheme 2).

As can be seen (Table 2, entries **9**–**13**), the *N*-Boc protection process was quite satisfactory because it could be quantitatively converted to its *N*-Boc esters of *α*-amino acids.

It was quite interesting to observe the *N*-Boc protection of many of the substrates gave optically pure *N*-Boc derivatives (as determined by optical rotation and comparison with literature values).

As can be seen from results in Table 2, the isolated yield of 9a–13a were in the range of 92–96%, the reaction could be completed in 5 min and 12 min.

**Scheme 3:**
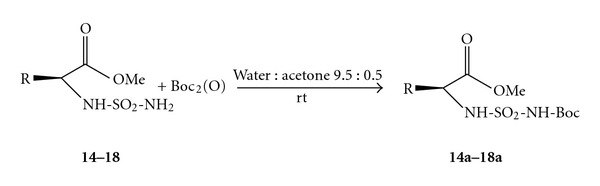


Encouraged by these experimental results, we extended our studies to series carboxylsulfamides aminoester derivatives (Scheme 3, Table 3).

The preparation of sulfamides amino-esters derivatives (**14–19**) was performed in four steps starting from amino acids (Gly, Ala, Val, Leu, and Phe) and chlorosulfonyl isocyanate (CSI) and *tert-*butanol after four steps: esterification-sulfamoylation, carbamoylation, and deprotection previously described [34].

The *N*-Boc protection reaction was studied using compounds **14**–**18 **as substrates in the same conditions. 1.0 mmol was treated with (Boc)_2_O 260 mg, 1 mmol in water : acetone at room temperature (Table 3, entries 14–18). The reaction was monitored by TLC. In most of cases, the desired product was obtained in good at excellent yields (Scheme 3).

The reaction preserves stereochemical integrity of amino esters derivatives. The reactions were rapid with most of the sulfamides studied (5–10 min) and were compatible with diverse sulfamides.

The propriety of this method can be formative of the application in the organic synthesis and particularly in peptide synthesis.

**Scheme 4:**
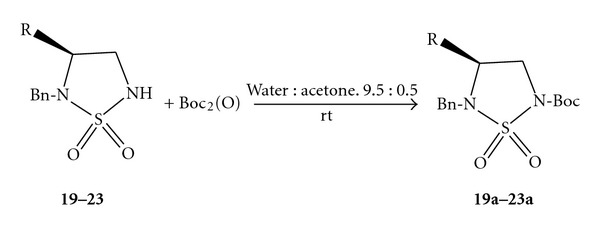


To explore the scope and limitations of this reaction, we extended our study the *N*-protection of cyclosulfamides (Scheme 4).

The synthesis of the cyclosulfamides (entries **19**–**23**) was achieved starting from CSI, and amino acids (Gly, Ala, Val, Leu, and Phe) according a general procedure previously described [34]. The derivatization of amino acids allowed the introduction of an alkyl group on C-4 well-defined configuration.

The cyclosulfamides (Table 4, entries** 19**–**23**) were tested under the same conditions of present protocol. The reaction is monitored by TLC, which indicates complete disappearance of (**19–23**) within 8 min at room temperature and atmospheric pressure, to afford the corresponding *N*-protected cyclosulfamides (**19a–23a**) with excellent yields.

N-Boc chiral cyclosulfamides entry (**19**–**23**) gave optically *N*-t-Boc derivatives (as determined by optical rotation and HPLC). In all cases, the *N*-protected cyclosulfamides (**19a**–**23a**) were less polar than his precursor (TLC).

**Scheme 5:**
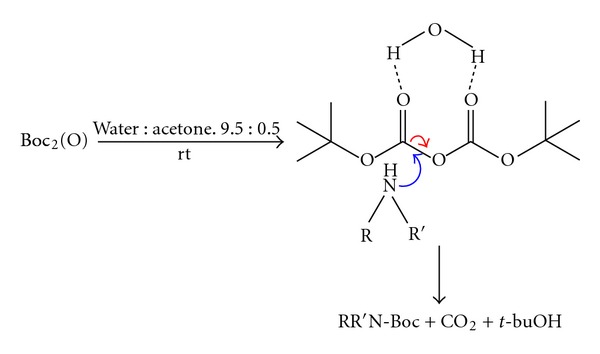
Electrophilic activation of Boc_2_(O) during water-mediated catalyzed the *N*-Boc formation from amines.

To explore the mechanism of these processes, we assume that hydrogen bond formation between water and the carbonyl oxygen atom of (Boc)_2_O causes electrophilic activation of the carbonyl group which make more susceptible to nucleophilic attack. Intramolecular nucleophilic attack by the nitrogen atom on the carbonyl carbon activated followed by release of CO_2_, t-BuOH, H_2_O and forms the carbamate (Scheme 5). 

The structures of all the compounds were unambiguously confirmed by usual spectroscopic methods. For the final derivatives, the different NMR spectra showed a signal of NH proton and appearance of signal corresponding to the *tert*-butyl protons. These compounds exhibited characteristic absorption in the IR spectrum with the absorption at 1702–1712 cm^−1^ (C=O). 

## 3. Conclusions

 In summary, we have developed a novel and efficient route for water-mediated *N*-*tert*-butoxycarbonylation of amines at room temperature. The absence of acid/base and the use of water makes present procedure environmentally friendly. We are exploring the protection of various diverse amines with other protecting groups applications and will report the finding in due course.

## 4. Experimental Section

All commercial chemicals and solvents were without further purification. All reactions were carried out under inert argon atmosphere. Melting points were determined in open capillary tubes on a Büchi apparatus and are uncorrected. 1H and 13C NMR spectra were recorded in a 250 MHz Brücker spectrometer. Microanalysis was performed in the microanalysis laboratory of ENSCM (Montpellier). Chemical shifts are reported in *δ* units (ppm) with TMS as reference. All coupling constants *J* are reported in Hertz. Multiplicity is indicated as s (singlet), d (doublet), t (triplet), q (quartet), m (multiplet), and combination of these signals. Electron Ionisation mass spectra (30 eV) were recorded in positive or negative mode on a Water MicroMass ZQ. High-resolution mass spectra were measured on a Jeol SX102 mass spectrometer and recorded in FAB positive mode. All reactions were monitored by TLC on silica Merck 60 F254 precoated aluminium plates and were developed by spraying with ninhydrin solution. Optical rotations were measured on a JUSCO DIP-370 digital polarimeter. Columns chromatographies were performed on Merck silica gel (230–400 mesh). Compounds 1–9 are available commercially N2-Boc-4- alkyle-N5-benzyl-1,2,5thiadiazolidine 1,1-dioxide (**1–5**).

The synthesis of the compounds, starting from (CSI) chlorosulfonyl isocyanate *tert*-butyl alcohol and methyl esters of amino acids (glycine, L-alanine, L-leucine, and L-phenyalanine) has been previously reported [34].

**N-Boc Protection: General Procedure**

In a 50 mL round flask with 9.5 mL of distilled water and 0.5 mL acetone, 1 mmol of amine was added, the mixture was stirred at room temperature for the few minutes.

Dichloromethane was added (5 mL), and the mixture was stirred. Progress of the reaction is monitored by TLC, which indicates complete disappearance of precursors amines. The organic layer was dried over anhydrous Na_2_SO_4_ and concentrated in vacuum. The residue was purified by column chromatography on silica gel with (CH_2_Cl_2_/MeOH, 9 : 1) to afford the *N*-Boc amines derivatives in high yields.

The synthesis of the compounds **14**–**23** has been previously reported for our research group [35, 36].

**(S)-Methyl 2-((N-(Tert-Butoxycarbonyl)Sulfamoyl)Amino)-3-Methylbutanoate 16a**

(Yield 92%); *R*_*f*_ = 0.72 (CH_2_Cl_2_-MeOH, 9.1), (mp 89–90°C), [*α*]_D_ = +2.5 (c = 1, EtOH), (KBr) *v*, cm^−1^: 1752 and 1697 (C=O), 1352 and 1158 (SO_2_); 3332, 3258 and 3274 (NH), 2964 (CH). ^1^H NMR (CDCl_3_): *δ* 7.20 (s, H, N**H**-Boc), 5.75 (d, *J* = 8.3 Hz, 1H, NH), 3.90 and 3.95 (dd, *J* = 4.8 and *J *′= 4.8 Hz, 1H, C*H); 3.78 (s, 3H, OCH_3_); 2.20 (m, 1H, 3H, CH *i*Pr); 0.90 and 1.10 (2d, *J* = 6.8 Hz, 6H, 2CH**_3_**), 1.45 (s, 9H, *t*-Bu). ^13^C NMR spectrum (125 MHz, CDCl_3_): *δ*, ppm (*J*, Hz): 19.70, 20.00, 27.56, 30.30, 56.70, 62.20, 84.34, 153.56,175.60.

Mass Spectrum (ESI^+^, 30 eV), *m/z* (*I*_*rel*⁡_, %): 311 [M+H]^+^ (100). Found, %: C, 34.42; H, 6.71; N, 13.12. C_6_H_14_N_2_O_2_S. Calculated, %: C, 34.28; H, 6.66; N, 13.33.

**(S)-Methyl 2-((N-(Tert-Butoxycarbonyl)Sulfamoyl)Amino)-4-Methylpentanoate 17a**

(Yield 95%); *R*_*f*_ = 0.67 (CH_2_Cl_2_-MeOH, 9.1), (mp 67-68°C), [*α*]_D_ = −14.5 (c = 1, MeOH), (KBr) *v*, cm^−1^: 1751 and 1698 (C=O), 1358 and 1162 (SO_2_); 3310 and 3251 (NH), 2987 (CH). ^1^H NMR spectrum (250 MHz, CDCl_3_): *δ*, ppm (*J*, Hz): 7.25 (s, H, N**H**-Boc), 5.20 (s, 1H, NH exch), 4.25 (t, *J* = 7.4, 1H, C*H); 3.66 (s, 3H, OCH_3_); 1.85 (m, 1H, *i*Pr), 1.55 (m, 2H, CH_2*β*_); 1.48 (s, 9H, t-Bu), 0.93 and 0.75 (2d, *J* = 2.9, 6H, 2CH_3_). ^13^C NMR spectrum (125 MHz, CDCl_3_): *δ*, ppm (*J*, Hz): 21.32, 22.73, 24.38, 27.52 41.40, 52.77, 54.74, 84.54, 152, 20, 174.79.

Mass Spectrum (ESI^+^, 30 eV), *m/z* (*I*_*rel*⁡_, %): 325 [M+H]^+^, (100).

Found, %: C, 37.46; H, 7.13; N, 12.54. C_7_H_16_N_2_O_2_S: Calculated, %: 37.50; H, 7.14; N, 12.50.

**(S)-Methyl 2-((N-(Tert-Butoxycarbonyl)Sulfamoyl)Amino)-3-Phenylpropanoate 18a**

(Yield 95%); *R*_*f*_ = 0.68 (CH_2_Cl_2_-MeOH, 9.1), (mp 131-132°C), [*α*]_D_ = + 12 (c = 1, MeOH), IR (KBr, *ν* cm^−1^): 1745 and 1702 (C=O), 1338 and 1152 (SO_2_); 3312, 3245, 3482, (NH). ^1^H NMR spectrum (250 MHz, CDCl_3_): *δ*, ppm (*J*, Hz): 7.25 (s, H, N**H**-Boc), 7.25 (m, 5H, Ar-H), 7.10 (s, H, N**H**-Boc), 5.60 (d, 1H, *J* = 8.8 Hz, NH), 4.90 (s, 2H, NH_2_), 4.40 (dt, *J* = 5.5 Hz and *J*′ = 8.8 Hz, 1H, C*H); 3.65 (s, 3H, OCH_3_); 3.00 and 3.20 (2dd, (ABX system) ^1^ *J* = 5.7, ^2^ *J* = 7.00 and *J*gem = 13.8, 2H, CH_2_), 1.45 (s, 9H, *t*-Bu). ^13^C NMR spectrum (125 MHz, CDCl_3_): *δ*, ppm (*J*, Hz): 27.45, 39.50, 52.50, 58.60, 84, 67, 127.70, 129.80, 129.90, 137.30, 150.00, 173.50. Mass Spectrum (ESI^+^, 30 eV), *m/z* (*I*_*rel*⁡_, %): 359 [M+H]^+^ (100). Anal. Calcd for C_10_H_14_N_2_O_2_S; C,46.51; H, 5.42; N, 10.85. Found; C, 46.49; H, 5.39; N, 10.80.

**(R)-Tert-Butyl 5-Benzyl-4-Isopropyl-1,2,5-thiadiazolidine-2-Carboxylate 1,1-Dioxide 21a**

(Yield 96%); *R*_*f*_ = 0.72 (CH_2_Cl_2_-MeOH, 95-5); (mp 82–84°C), [*α*]*_D_*= +5 (c = 1, EtOH). (KBr) *v*, cm^−1^: 3331 and 3314 (NH); 1345 and 1165 (SO_2_), 1708 (CO). ^1^H **N**MR spectrum (250 MHz, CDCl_3_): *δ*, ppm (*J*, Hz): 7.40 (m, 5H, ArH); 4.35 (d, *J* = 13.8 Hz, 1H, CH_2_-Ph); 3.95 (d, *J* = 13.8 Hz, 1H, CH_2_-Ph); 3.40 (m, 3H, *CH and CH_2_); 2.8 (m, 1H, CH *i*Pr); 1,58 (s, 9H, *t*-Bu), 0.90 and 1.00 (2d, *J* = 6.7 Hz, 6 H, 2CH_3_). ^13^C NMR spectrum (125 MHz, CDCl_3_): *δ*, ppm (*J*, Hz): 166.65, 139.2, 128.3, 129.4, 12.5, 84, 52, 51.2, 50.6, 32.3, 27, 42, 23.5, 19.4, 18.2.

Mass Spectrum (ESI^+^, 30 eV), *m/z* (*I*_*rel*⁡_, %): 355. [M+H]^+^ (72), 91 [Bn]^ +^ (80).

Anal. For C_12_H_18_N_2_O_2_S Calcd: C, 56.69; H, 7.08; N, 11.02. found: C, 56.67; H, 7.14; N, 10.95.

**(R)-Tert-Butyl 4,5-Dibenzyl-1,2,5-thiadiazolidine-2-Carboxylate 1,1-Dioxide 23a**

(Yield = 93%); *R*_*f*_ = 0.52 (CH_2_Cl_2_); (mp 97-98°C),[*α*]*_D_* = −23° (c = 1, EtOH). (KBr) *v*, cm^−1^: 3269 (NH); 1338 and 1172 (SO_2_). ^1^H NMR spectrum (250 MHz, CDCl_3_): *δ*, ppm (*J*, Hz): 7.52 (m, 10H, ArH); 4.40 (m, 1H, CH_asy_); 4.10 (d, *J* = 13.6, 1H, CH_2_-Ph); 4.35 (d, *J* =13.6, 1H, CH_2_-Ph); 2.90 (m, 2H, CH_2_); 3.50 and 3.20 (2dd, *J* = 18.3, ^1^ *J* = 4.7 and ^2^ *J*= 7.3, Hz, 2H, CH_2_-Ph), 1.45 (s, 9H, t-Bu). ^13^C NMR spectrum (125 MHz, CDCl_3_): *δ*, ppm (*J*, Hz): 138,7, 137.3, 129.2, 128.3, 127.5, 127.1, 125.5, 124.5, 57.3, 54.2, 52.1, 42.7.

Mass Spectrum (ESI^+^, 30 eV), *m/z* (*I*_*rel*⁡_, %): 303 [M+H]^+^ (100), 91 [Bn]^ +^ (67).

Found, %: C, 63.51; H, 5.92; N, 09.29. C_16_H_18_N_2_O_2_S. Calculated, %: C, 63.57; H, 5.96; N, 09.27.

## Figures and Tables

**Table 1:** *N*-Boc protection of amines derivative^a^.

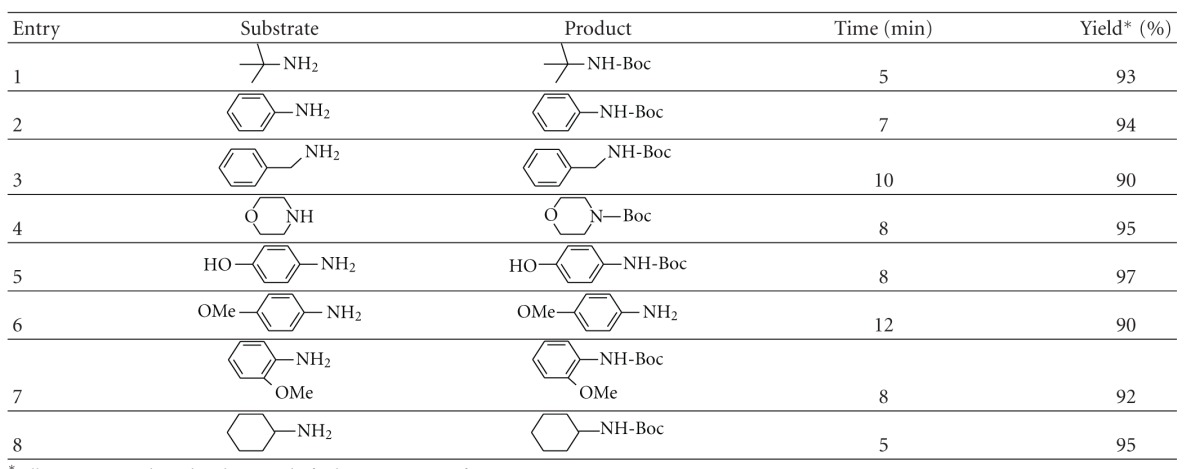

*All reactions conducted with 1 mmol of substrate in 1 mL of water : acetone 9.5 : 0.5.

*Isolation yield after purification.

**Table 2:** *N*-Boc protection of aminoesters^a^.

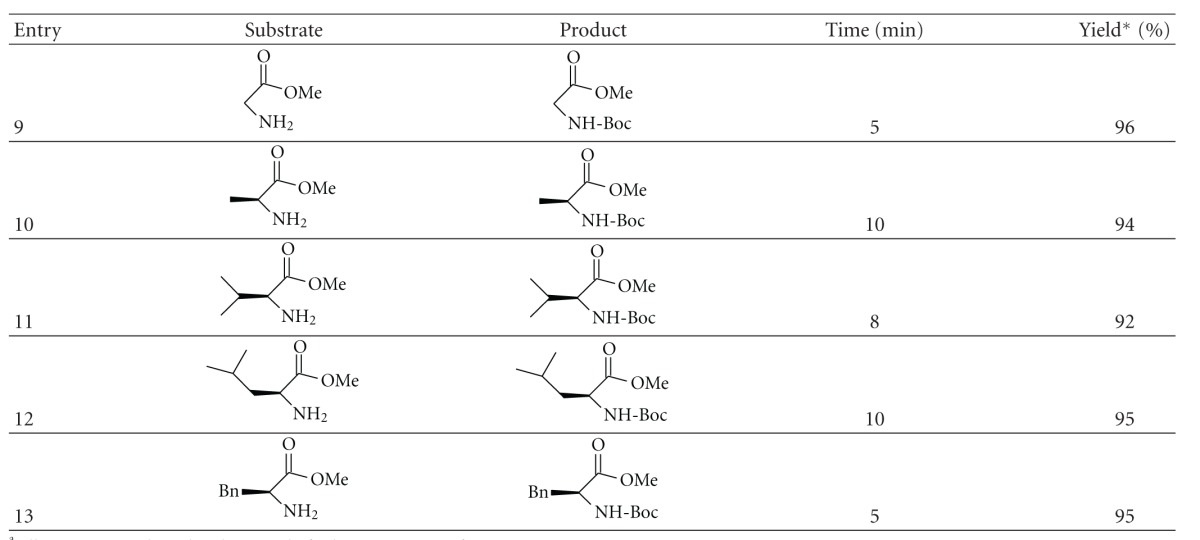

^a^All reactions conducted with 1 mmol of substrate in 1 mL of water: aceton 95 : 5.

*Isolation yield after purification.

**Table 3:** *N*-Boc protection of linear carboxylsulfamides^a^.

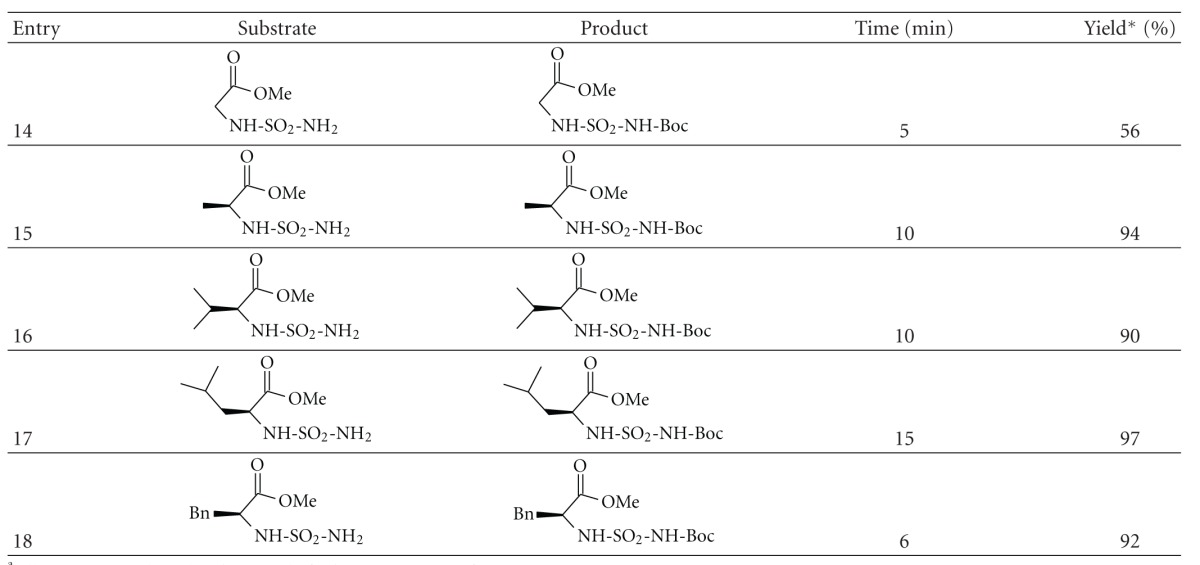

^a^All reactions conducted with 1 mmol of substrate in 10 mL of water : acetone 9.5 : 0.5.

*Isolated yield after purification.

**Table 4:** *N*-Boc protection of cyclosulfamides^a^.

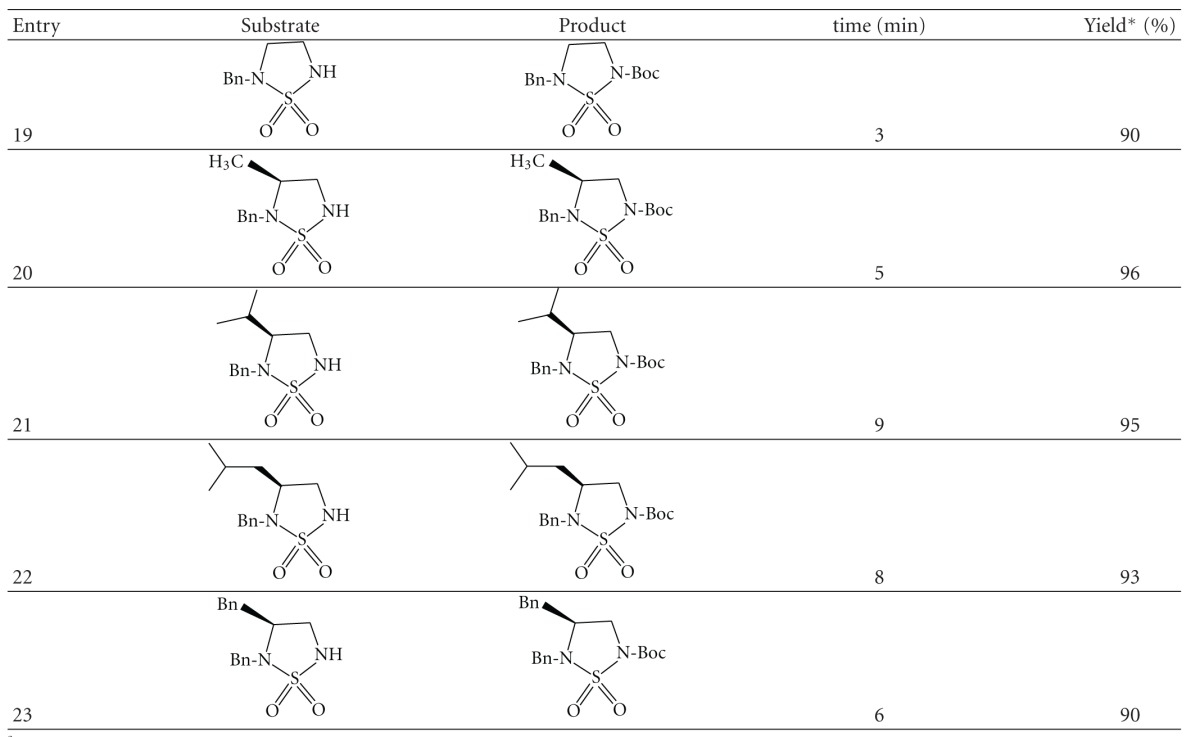

^a^All reactions conducted with 1 mmol of substrate in 10 mL of Water : acetone 9.5 : 0.5.

*Isolated yield after purification.
